# Patterns of traumatic brain injury and six-month neuropsychological outcomes in Uganda

**DOI:** 10.1186/s12883-019-1246-1

**Published:** 2019-02-04

**Authors:** Paul Bangirana, Bruno Giordani, Olive Kobusingye, Letisia Murungyi, Charles Mock, Chandy C. John, Richard Idro

**Affiliations:** 1 Department of Psychiatry, Makerere University, Kampala, Uganda; 2 Department of Psychiatry, University of Michigan, Ann Arbor, MI USA; 3 Trauma, Injury, and Disability Track, School of Public Health, Makerere University, Kampala, Uganda; 4 Global Health Uganda, Kampala, Uganda; 5 Harborview Injury Prevention & Research Center, University of Washington, Seattle, WA USA; 6 Department of Paediatrics, Indiana University, Indianapolis, IN USA; 7 Department of Paediatrics and Child Health, Makerere University, Kampala, Uganda

**Keywords:** Cognition, Brain injury, Anxiety, Depression, Physical disability, Traffic crashes

## Abstract

**Background:**

Traumatic brain injuries in Uganda are on the increase, however little is known about the neuropsychological outcomes in survivors. This study characterized patients with traumatic brain injury (TBI) and the associated six-month neuropsychological outcomes in a Ugandan tertiary hospital.

**Methods:**

Patients admitted at Mulago Hospital with head injury from November 2015 to April 2016 were prospectively enrolled during admission and followed up at six months after discharge to assess cognition, posttraumatic stress symptoms (PTSS), depression symptoms and physical disability. The outcomes were compared to a non-head-injury group recruited from among the caretakers, siblings and neighbours of the patients with age and sex entered as covariates.

**Results:**

One hundred and seventy-one patients and 145 non-head injury participants were enrolled. The age range for the whole sample was 1 to 69 years with the non-head injury group being older (mean age (SD) 33.34 (13.35) vs 29.34 (14.13) years of age, *p* = 0.01). Overall, motorcycle crashes (36/171, 38.6%) and being hit by an object (58/171, 33.9%) were the leading causes of TBI. Head injury from falls occurred more frequently in children < 18 years (13.8% vs 2.8%, *p* = 0.03). In adults 18 years and older, patients had higher rates of neurocognitive impairment (28.4% vs 6.6%, *p* < 0.0001), PTSS (43.9% vs 7.9%, *p* < 0.0001), depression symptoms (55.4% vs 10%, p < 0.0001) and physical disability (7.2% vs 0%, *p* = 0.002). Lower Glasgow Coma Score (GCS) on admission was associated with neurocognitive impairment (11.6 vs 13.1, *p* = 0.04) and physical disability (10 vs 12.9, *p* = 0.01) six months later.

**Conclusion:**

This first such study in the East-African region shows that depth of coma on admission in TBI is associated with neurocognitive impairment and physical disability.

## Background

Traumatic brain injury (TBI) is a leading cause of death and disability in young people worldwide, most especially in low and middle income countries [1–3]. In low and middle income countries, road traffic crashes are the leading causes of TBI in young people while falls are the leading causes in the elderly [2]. In 2013, there were 1.25 million deaths from road traffic crashes worldwide with the African region having the highest fatality rate [4]. In addition, the risk of dying from road traffic crashes was highest in Africa (26.6 per 100,000 population), and lowest in the European region (9.3 per 100,000). Between 2010 and 2013, low and middle income countries saw an increase in road traffic deaths while high income countries reported a general decrease [4]. Over the last two decades, there has been an increase in the contribution of road traffic crashes to deaths globally [5]. At the same time, road traffic crashes moved from being the 12th leading cause of disability adjusted life years (DALYs) to the 10th [5].

Road traffic crashes in Uganda contribute between 27 to 60% of the patients in rural and urban health centres [6–8]. In addition, mortality from injuries is highest among road traffic crashes [6]. Most of the traffic crashes involve motorcycle riders (73%), a common mode of transport in the country [7]. Over the years, motorcycles have been involved in an increasing percentage of total crashes, moving from 4.7% in 1979 [9], to 25% in 2001 [10] to over 50% in 2008 [7, 11]. This changing pattern highlights the important contribution of motorcycle traffic to injuries in Uganda.

Most head injuries (74.6%) presenting to hospital in Uganda have abnormal computed tomography (CT) images manifesting as extra-cerebral and subdural hemorrhages [12]. Two studies have described the immediate outcomes of brain injury in Uganda. In one study at Mulago Hospital, open head injuries were associated with poor outcome with a mortality rate of 10.5% [8]. The majority (73%) had good outcome, 13.5% had moderate disability, 2.1% had severe disability and 0.3% were in a vegetative state. The other study found a mortality rate of 9% and the factors associated with death were low Glasgow coma scores, intracranial hemorrhage and skull fractures [12]. None of these two studies described the longer-term outcomes of brain injury, which are determinants for quality of life. Indeed, Erem recommended at least 6 months of follow-up for brain injury survivors in order to document the persisting outcomes [12] though this time-point may be too early to predict persistent outcomes as substantial recovery might take place after 6 months [13, 14].

Brain injuries in children and adults are associated with work and school problems, poor cognition and overall poor quality of life [15–19]. Understanding the neuropsychological outcomes of TBI can inform rehabilitation needs. In high-income countries, neuropsychological test performance at five months post-injury is associated with return to productivity in adults [20]. In another study, when neuropsychological, emotional and behavioral scores were considered in a model, only neuropsychological scores were predictive of functional outcome at 12 months post injury [21]. Characterizing TBI and the associated neuropsychological outcomes in Uganda and the surrounding region has not been done to our knowledge. This is especially important in Uganda that lacks adequate pre-hospital trauma care compared to high-income countries [22] that can reduce mortality and post-discharge morbidity. In addition, only 25% of the hospitals and health centres in Uganda have good capacity to offer emergency care and surgical services [23] which may affect TBI outcomes. Without neuropsychological assessment, it is difficult to predict recovery in patients and or develop rehabilitation tailored to a patient’s disability. The present study was designed to characterize cases of TBI and assess neurocognitive function, posttraumatic stress disorder symptoms (PTSS), depression symptoms and physical disability six months later in Uganda.

## Methods

### Study design and participant selection

This prospective cohort was recruited between November 2015 and April 2016 and followed up at six months. Patients with TBI were recruited from Mulago National Referral Hospital which serves Kampala, the capital city of Uganda, and the surrounding districts. Patients were recruited from the Neurosurgical ward during admission. Inclusion criteria for the patients were; a) admitted with TBI as per the admission diagnosis during the course of the study and able to give consent, b) admitted with TBI before the start of the study and were still admitted by the start of the study and able to give consent and, c) severely ill patients initially unable to give consent but recovered during the study and were able to give consent. Patients too injured to give consent throughout the course of their admission were excluded. Caretakers, unexposed siblings and neighbours of the patients were recruited as a non-head injury group. After recruitment, patients were interviewed to get a medical history while other information was abstracted from the hospital files and an appointment given for the assessments after six months. The non-head injury group subjects were consented and had the medical history interview and assessments done on the same day.

### Assessments

#### Neurocognitive functioning

The Cogstate computer-administered neuropsychological test battery was used to assess neurocognitive functioning in participants 5 years and older [24]. It has been validated for use in Ugandan children and is used in clinical trials locally [25–27]. Though not validated in Ugandan adults, it has been widely used in assessing adults after TBI elsewhere and has sound psychometric properties in this population [28, 29]. The test assesses psychomotor speed, visuo-motor function, visual attention, visual learning and memory, working memory and spatial problem solving. It employs non-culturally biased maze and card tasks to assess these abilities, thus can be used in non-English speaking settings [26, 27]. Psychomotor speed is measured by the Detection task, where the participant is directed to press the YES button as soon as the card turns over. Visuo-motor function was assessed by the Chase task using a 10 × 10 grid of tiles, where the subject chases a target moving through the maze by tapping the tiles it is on. Visual attention was measured by the Identification task, where the subject presses the YES button when a red card turns over and NO if it is not red. Visual learning and memory were measured by the One Card Learning task, where the subject presses the YES button if the card displayed has been seen before during the task or NO if it has not. Working memory was measured by the One Back task, where the subject presses the YES button if the card displayed is the same as the previous card task or NO if it is not. Spatial problem solving was measured using the Maze Learning task, where the subject through trial and error taps on the tiles to discover a hidden path beneath while receiving feedback. This task measures aspects of executive function that includes spatial working memory, error monitoring [28].

#### PTSS

PTSS was assessed using the PTSD checklist-civilian version (PCL), based on the DSM-IV criteria [30]. It has 17 items corresponding to the criteria B, C and D of the DMS-IV on a five point Likert scale. An item is considered present when it has a score of three or higher on the Likert scale. It has test-retest reliability of 0.92 for immediate retesters and 0.68 at two weeks [31]. When considering a cut-off score of 44 and above, the PCL has a sensitivity of 90% and specificity of 95% [31]. Participants with one intrusive symptom from criteria B, three from criteria C and two from criteria D as well as having a total score of 44 and above were also considered to have PTSS. This mixed method that was used in the present study employing cutoff scores and following the DSM criteria increases the diagnostic efficiency of PTSD to 0.96 [31].

#### Depression

The Self Reporting Questionnaire was used to assess depression symptoms [32]. It is comprised of 20 items with Yes/No responses which have been used to screen for depression across multiple cultures [33, 34]. A cut-off score of ≥6 was used to determine those with depression symptoms, based on a validation study of the Self Reporting Questionnaire in Uganda [34]. This cut-off score of ≥6 had a sensitivity of 84% and specificity of 93% in the above Ugandan study [34].

#### Physical disability

The Functional Independence Measure (FIM) was used to measure the patient’s burden of care [35]. It assesses activities of daily living in the physical and cognitive domains using 18 items. They are scored from 1 to 7, with 1 indicating total dependence and 7 total independence. Scores of the 13 items assessing physical ability were used as a specific measure of physical disability in this study. FIM scores have been shown to be predictive of employment status three years after TBI [36].

### Analyses

Cogstate raw scores were converted into age adjusted Z scores as previously described [37]. Based on conventional practice for diagnosing neurocognitive impairment in adults and children internationally and in local studies [37, 38], a score of ≤2 standard deviations below the expected mean was categorized as impaired in a domain. Categorical comparisons between groups were done using Chi-Square test with Fisher’s exact test where appropriate. T-test was used to compare the association between continuous clinical and sociodemographic variables and categorical outcomes of the study. Analysis of covariance was used to compare test outcomes between the TBI and non-head injury groups controlling for age and sex.

## Results

### Participant’s characteristics and patterns of injury

One hundred and seventy-one patients with TBI were recruited together with 145 non-head injury group participants. The patients were predominantly male compared to the non-head injury group (83% vs 53.1%, *p* < 0.0001) and were younger (mean age (SD) 33.34 (13.35) vs 29.34 (14.13) yrs., *p* = 0.01) (Table 1).

**Table 1:** Sociodemographic characteristics of the participants

	Cases (*n* = 171)	Controls (*n* = 145)	*p*
Age, years, M (SD)	29.34 (14.13)	33.34 (13.35)	0.01^a^
Above 18 years N(%)	142 (83%)	140 (96.6)	< 0.0001^b^
Sex, Male N(%)	142 (83%)	77 (53.1%)	< 0.0001^b^

^a^ T- test

^b^ Chi square test

Motor cycle crashes and being hit by an object on the head were the commonest causes of TBI (38.9 and 33.9% respectively) with sports injuries being least common (1.2%). Falls were more common in children below 18 years than in adults (13.8% vs 2.8%, *p* = 0.03). The duration of admission ranged from 1 day to 55 days with a mean duration of 4.93 (Table 2). Most injuries were closed head injuries (67.1%). CT scans were done in 142 patients with 104 (73.6%) showing brain or cranial trauma. Self-reported use of a helmet among riders was associated with a better GCS score (14.2 vs 12.4, *p* = 0.009). Of the 171 patients, only 96 (56.5%) returned for assessment six months later of which 13 were below 18 years and are not included in the neuropsychological outcomes below. Of the 75 who did not return, 51 (68%) were lost to follow-up (did not return phone calls, had no phone contacts), 16 (21.3%) withdrew from the study, 6 (8%) died from complications of the injury and 2 (2.7%) were too severely injured to move.

**Table 2:** Patterns of brain injury across the lifespan in Uganda

Variable	N (%)
Age in years, M (SD), range	29.34 (14.13), 1.16–68.72
GCS score, M (SD), range	12.91 (2.48), 6–15
Duration of admission in days, M (SD), range	4.93 (5.85), 1–55
Cause of injury	
Motor vehicle accident	29 (17)
Motorcycle accident	66 (38.6)
Hit by object	58 (33.9)
Bicycle	8 (4.7)
Fell down	8 (4.7)
Sports injury	2 (1.2)
Nature of injury	
Open head injury^a^	56 (32.9)
Closed head injury	114 (67.1)
CT scan showing trauma^b^	106 (73.6%)
Accident victim status	
Driver	3 (1.8)
Rider	59 (34.5)
Passenger	24 (14)
Pedestrian	66 (38.6)
Safety precaution	
Helmet use among riders (*n* = 59)	18 (30.5)
Under alcohol influence (n = 171)	16 (9.4)

All figures are N (%) unless otherwise stated

^a^1 non-response whether injury was open or closed

^b^CT scan results available for 144 out of 171 cases

### Neuropsychological outcomes of TBI at six months

Neuropsychological results are presented for the 81 patients aged 18 years and above who returned at 6 months and 137 participants in the non-head injury group who were 18 years and above. Patients had a higher rate of neurocognitive impairment (having impairment in any of the neurocognitive domains tested) than the non-head injury group (28.4% vs 6.6%, *p* < 0.0001). Of the six neurocognitive domains tested, patients had significantly higher rates of impairment in three (psychomotor speed, visual attention and working memory) with visual attention having the highest rate (16% vs 2.2, p < 0.0001) (Table 3). 7.2% of the patients had physical disability compared to 0% of the non-head injury group (*p* = 0.002). High rates of PTSS and depression symptoms were observed in the patients compared to the non-head injury group (43.9% vs 7.9%, *p* < 0.0001 and 55.4% vs 10%, p < 0.0001 respectively).

**Table 3:** Frequency of impairment in test outcomes between the groups

Domain	Cases (*n* = 81)	Controls (*n* = 137)	*P*
Psychomotor speed	8 (9.9)	1 (0.7)	0.002^1^
Visual motor	1 (1.2)	1. (0.7)	0.61^1^
Visual attention	13 (16)	3 (2.2)	< 0.0001
Visual learning and memory	1 (1.2)	1 (0.7)	0.61^1^
Working memory	6 (7.4)	0 (0)	0.002^1^
Spatial problem solving	4 (4.9)	4 (2.9)	0.34^1^
Any cognitive impairment	23 (28.4)	9 (6.6)	< 0.0001
Had physical disability^a^	6 (7.2)	0 (0)	0.002^1^
Had PTSS^b^	36 (43.9)	11 (7.9)	< 0.0001
Had Depression symptoms^c^	46 (55.4)	14 (10)	< 0.0001

All figures are N (%)

^1^Fisher’s exact test

^a^Cases *N* = 83, Controls *N* = 140

^b^Cases *N* = 82, Controls *N* = 139

^c^Cases N = 83, Controls *N* = 140

A lower coma score was associated with neurocognitive impairment (11.6 vs 13.1, *p* = 0.04) and physical disability (10 vs 12.9, *p* = 0.01). No other demographic or clinical factors (e.g. nature of the injury or CT scan findings) were associated with the study outcomes. Analysis of the continuous outcomes produced similar results as the above categorical ones except in working memory and visual motor (Table 4).

**Table 4:** Estimated mean differences of test outcomes between the groups

Domain	Cases (n = 81)	Controls (n = 137)	Mean difference	*P* ^*1*^
Psychomotor speed	−0.45 (0.12)	0.03 (0.09)	− 0.48 (0.15)	0.001
Visual motor	−0.76 (0.11)	0.05 (0.09)	−0.81 (0.15)	< 0.0001
Visual attention	−0.83 (0.12)	0.02 (0.09)	−0.85 (0.16)	< 0.0001
Visual learning and memory	0.06 (0.11)	0.04 (0.09)	0.02 (0.14)	0.91
Working memory	−0.27 (0.12)	0.04 (0.09)	−0.30 (0.15)	0.05
Spatial learning	0.04 (0.17)	−0.01 (0.13)	0.05 (0.21)	0.82
Physical disability^a^	89.24 (0.40)	91.00 (0.30)	−1.76 (0.51)	< 0.001^2^
PTSS^b^	40.94 (1.43)	22.60 (1.08)	18.34 (1.83)	< 0.0001^2^
Psychological Distress^c^	7.07 (0.41)	1.72 (0.31)	5.35 (0.52)	< 0.0001^2^

All figures are estimated means (standard error)

^1^Means are age adjusted Z scores with sex entered as a covariate

^2^Means are raw scores with sex and age entered as covariates

^a^Cases N = 83, Controls *N* = 140

^b^Cases N = 82, Controls *N* = 139

^c^Cases *N* = 83, Controls *N* = 140

## Discussion

This paper is the first to characterize TBI in Uganda and the associated extended neuropsychological outcomes. Motorcycle crashes and being hit with an object on the head were the main causes of TBI. Self-reported helmet use among riders was at 30.5% and this was associated with a higher GCS score on admission. Physical disability was the least common outcome at 7.2% while PTSS and depression symptoms were at 43.9% and 55.4% respectively. Neurocognitive impairment was experienced by 28.4%. Low GCS score was associated with physical disability and neurocognitive impairment.

In a prospective cohort study assessing cognitive outcome one year after TBI, Sigurdardottir et al. found 67% of the patients were cognitively impaired with processing speed and memory being the most affected [18]. Skandsen and colleagues found a lower rate of 43% cognitively impaired after TBI with information processing and verbal memory mainly affected [19]. These studies have higher rates of cognitive impairment than we observed likely due to a select sample in our study that had less severe injuries, were functional and able to travel to hospital for assessment. Despite these high rates of cognitive impairment, improvement in some cognitive domains from three to 12 months has been observed when patients were classified according TBI severity [39]. However, the 28.4% with cognitive impairment in our study is significant necessitating appropriate cognitive rehabilitation. This service is currently not available at the tertiary hospital though experimental cognitive rehabilitation studies have been carried out among children in the same setting [25, 27].

There were notable differences in the causes of TBI and demographics of the victims between Uganda and the Western world. The main causes of TBI in the US are falls and motor vehicle crashes while in our study it was motorcycle accidents and being hit on the head [1]. Bicycles and motorcycles are the commonest forms of transport owned by Ugandans accounting for high rates of motorcycle accidents [40]. Uganda has in the recent years seen a new wave of iron-bar robbers who often hit their victims on the head [41]. These patients have been increasing despite increased police presence, explaining the high number of injuries caused by being hit on the head [42]. A more recent report from the Uganda Bureau of Statistics shows motorcycle accidents having the largest proportion of death or serious injury from road traffic accidents [40]. Our findings of motor cycles being the main causes of TBI are similar to previous reports in Uganda [7, 11, 43]. CT scans showed trauma in 73.6% which is close to the 74.6% that was reported in an earlier study [12].

PTSS and depression symptoms were the commonest outcomes reported by 43.9% and 55.4% of the TBI patients respectively. PTSD after TBI has been reported in 2.9% to 58.8% of the survivors depending on the assessments used [44]. Use of questionnaires is associated with high PTSD rates of 59% compared to 3% when using a structured clinical interview [45]. These studies and our own that used checklists rather than a structured interview imply that PTSS rates in our sample could be an over estimate. Depression, anxiety, pain, lower education and re-integration into normal living have been associated with PTSS after traumatic brain injury in other studies [44]. Our study did not identify factors associated with PTSS, since only limited information could be obtained from the participants.

Rates of depression vary across TBI studies, with rates between 25% and 50% reported within the first year after injury [46]. In a checklist study, 53% of injury victims met criteria for depression within the first year [47], a rate closer to what we observed at 6 months using a checklist. The variation in depression rates across studies has been attributed to heterogeneity of the samples and different instruments used [46]. Use of checklists such as that employed in this study does yield a high percentage of individuals screening positive for depression compared to use of structured diagnostic interviews that result in lower depression rates e.g. [48]. Predictors of depression within a year after TBI include; a prior history of mental illness, positive screen for depression and amphetamines at the time of the injury and a history of alcohol dependence [49]. Determination of predictors of long-term outcomes can help in identifying vulnerable patients and instituting appropriate interventions [49]. These high rates of mental health problems among survivors highlight the need to have routine psychological assessment of TBI survivors at discharge and regular follow-up.

The association between helmet use among riders and coma score highlights the importance of safety precautions in preventing brain injury. Although helmet use was not associated with any outcome at six months, GCS score was associated with neurocognition and physical disability at 6 months. Coma duration has been associated with neurocognitive outcomes in other TBI and acquired brain injury studies [37, 50, 51]. Future studies should examine whether GCS scores are associated with persisting sequelae beyond the six months period used in this study.

This study had some methodological limitations that need to be considered when interpreting the results. The instruments used for depression and posttraumatic stress symptoms are not diagnostic and thus the rates presented may be different once formal diagnostic procedures are used. The follow-up period was short at 6 months, though longer than any previous study in Uganda. Outcomes are presented for only adults and maybe not be generalizable to pediatric populations. Only patients able to give consent during admission or at discharge were enrolled, thus severely injured patients were excluded resulting in a sample of not very severely injured participants as evidenced in low rates of physical disability in the study. This may therefore lead to underestimation of the neuropsychological outcomes of TBI in Uganda. There was a high loss to follow-up in the study with only 56.5% returning for the six months assessment. Participants who withdrew and those lost to follow-up could present a group with severe physical disability that limits their mobility, resulting in the present finding of a lower prevalence of physical disability. However, there was no difference in sociodemographic and clinical characteristics between those who returned and those who did not.

## Conclusion

High rates of neuropsychological impairment, depression symptoms and posttraumatic stress symptoms are common in Ugandans six months after TBI. Motorcycle crashes are a leading cause of TBI, and helmets which are associated with better coma outcomes, are not used by the majority of the riders. A low coma score was associated with physical disability and neurocognitive impairment. Public health efforts towards road safety practices (e.g., helmet use) need to be strengthened. Psychological assessment and interventions need to be integrated into the routine management of TBI in Uganda. Assessment can include brief computer or tablet-based neuropsychological assessments, such as the CogState system used in this study to monitor progress and determine cognitive rehabilitation needs. This can be done within six months of discharge and follow-up as deemed necessary.

## Abbreviations

CT: Computed Tomography
DALYs: Disability adjusted life years
DMS-IV: Diagnostic and Statistical Manual of Mental Disorders, fourth edition
FIM: Functional Independence Measure
GCS: Glasgow Coma Score
PCL: PTSD Checklist
PTSD: Posttraumatic Stress Disorder
PTSS: Posttraumatic Stress Symptoms
SD: Standard Deviation
TBI: Traumatic Brain Injury
